# Wertigkeit von Patient-Reported Outcome Measures zur Bewertung des Versorgungsvorteils der Sprachprozessorumversorgung bei Patient/-innen mit Cochleaimplantaten

**DOI:** 10.1007/s00106-023-01341-7

**Published:** 2023-08-04

**Authors:** Susen Lailach, Alexander Lenz, Thomas Zahnert, Marcus Neudert

**Affiliations:** Klinik und Poliklinik für Hals‑, Nasen‑, Ohrenheilkunde, Kopf- und Halschirurgie, Sächsisches Cochlear Implant Centrum, Universitätsklinikum Dresden, Fetscherstraße 74, 01307 Dresden, Deutschland

**Keywords:** Prothesen und Implantate, Hörtests, Sprachaudiometrie, Qualitätsverbesserung, Sprachverstehen, Prostheses and implants, Speech audiometry, Hearing tests, Quality improvement, Speech perception

## Abstract

**Hintergrund:**

Bei mit einem Cochleaimplantat (CI) versorgten Patient/-innen sollte alle 6 Jahre die Versorgung mit einem neuen Sprachprozessor geprüft werden. Ziel der Analyse ist die Erfassung des subjektiven und audiologischen Nutzens durch die Umversorgung.

**Methodik:**

Das Sprachverstehen und der subjektive Nutzen wurden von 99 Patient/-innen mit dem alten sowie dem neuen Sprachprozessor nach 4‑wöchiger Tragezeit analysiert. Das Sprachverstehen wurde mittels Freiburger Einsilbertest in Ruhe (FBE) bei 65 dB sowie 80 dB und Oldenburger Satztest (OlSa) bei 65 dB Störgeräusch und adaptivem Sprachschallpegel ermittelt. Zur Erfassung der subjektiven Hörbeeinträchtigung wurde der Abbreviated Profile of Hearing Aid Benefit (APHAB), zur Bewertung der subjektiven Zufriedenheit der Audio Processor Satisfaction Questionnaire (APSQ) herangezogen.

**Ergebnisse:**

Die Sprachprozessorumversorgung führte zu einer signifikanten Verbesserung des Sprachverstehens in Ruhe bei 65 dB (mittlere Differenz 8,9 ± 25,9 Prozentpunkte, *p* < 0,001) und 80 dB (mittlere Differenz 8,1 ± 29,7 Prozentpunkte, *p* < 0,001) sowie im Störgeräusch (mittlere Differenz 3,2 ± 10,7 dB S/N, Signal-Rausch-Abstand; *p* = 0,006). Anhand des APHAB konnte eine signifikante Verbesserung (mittlere Differenz 0,07 ± 0,16; *p* < 0,001) der Hörbeeinträchtigung in allen Hörsituationen nachgewiesen werden. Der APSQ zeigte eine signifikant höhere Zufriedenheit der Patient/-innen mit dem neuen Sprachprozessor (mittlere Differenz: 0,42 ± 1,26; *p* = 0,006). Bei vergleichender Bewertung des Versorgungsvorteils anhand subjektiver und sprachaudiometrischer Ergebnisse konnte ein Anteil von 35–42 % der Patient/-innen identifiziert werden, welcher subjektiv von der Umversorgung profitierte, jedoch keinen sprachaudiometrisch messbaren Versorgungsvorteil hatte.

**Schlussfolgerung:**

Es zeigte sich eine signifikante Verbesserung des audiologisch messbaren und subjektiv reflektierten Sprachverstehens sowie der Zufriedenheit der Patient/-innen nach Umversorgung. Bei Patient/-innen mit nur geringer Verbesserung des audiologisch messbaren Sprachverstehens sollte zusätzlich der subjektive Nutzen mit validierten Messinstrumenten erfasst werden, um gegenüber den Kostenträgern eine Umversorgung zu begründen.

## Abstract (en)

**Background:**

Patients with a cochlear implant (CI) should be evaluated for a new speech processor every 6 years. The aim of this analysis was to assess the subjective and audiological benefit of upgrades.

**Methods:**

Speech understanding and subjective benefit were analyzed in 99 patients with the old and the new speech processor after 4 weeks of wearing. Speech understanding was assessed using the Freiburg monosyllabic test in quiet (FBE) at 65 dB and 80 dB, and the Oldenburg Sentence Test (OLSA) at 65 dB noise with adaptive speech sound level. The Abbreviated Profile of Hearing Aid Benefit (APHAB) was used to assess subjective hearing impairment, and the Audio Processor Satisfaction Questionnaire (APSQ) was used to assess subjective satisfaction.

**Results:**

The speech processor upgrade resulted in a significant improvement of speech understanding in quiet at 65 dB (mean difference 8.9 ± 25.9 percentage points, *p* < 0.001) and 80 dB (mean difference 8.1 ± 29.7 percentage points, *p* < 0.001) and in noise (mean difference 3.2 ± 10.7 dB signal-to-noise ratio [S/N], *p* = 0.006). Using the APHAB, a significant improvement (mean difference 0.07 ± 0.16, *p* < 0.001) in hearing impairment was demonstrated in all listening situations. The APSQ showed significantly higher patient satisfaction with the new speech processor (mean difference 0.42 ± 1.26, *p* = 0.006). A comparative assessment of the benefit based on subjective and speech audiometric results identified a proportion of patients (35–42%) who subjectively benefited from the upgrade but had no measurable benefit based on speech audiometry.

**Conclusion:**

There was a significant improvement in audiologically measurable and subjectively reflected speech understanding and patient satisfaction after the upgrade. In patients with only a small improvement in audiologically measurable speech understanding, the subjective benefit should also be assessed with validated measurement instruments in order to justify an upgrade to the payers in the health sector.

Die Weiterentwicklung der extern getragenen Sprachprozessoren von Cochleaimplantaten (CI) unterliegt einem kontinuierlichen Prozess. Die Neuentwicklung eines Sprachprozessors fokussiert oft auf ergonomische Verbesserungen, wie z. B. eine geringere Prozessorgröße, eine verbesserte Benutzerfreundlichkeit durch verbesserte Bedienelemente oder Anschlussmöglichkeiten für externe Geräte [27]. Durch Optimierung der Sprachcodierungsstrategien, Signalvorverarbeitung und der Mikrofontechnologie können neue Sprachprozessoren auch zur Verbesserung der Hörleistung beitragen [4, 9, 21, 24]. Hierzu sind beispielsweise die Einführung neuer Richtmikrofonoptionen, aber auch Möglichkeiten der automatischen Adaptation an spezielle Hörsituationen oder die Rauschunterdrückung zu nennen [18–20, 25]. Viele dieser Funktionen wurden aus der modernen konventionellen Hörsystemversorgung übernommen. Die Verbindungsfähigkeit mit externen Geräten, wie Mobiltelefonen, Tablets usw. erlaubt es hörgeschädigten Patient/-innen zudem, den alltäglichen Kommunikationsanforderungen einfacher und besser gerecht zu werden [27]. Nach Zulassung neuer Sprachprozessoren werden diese regelhaft zu Standardprozessoren, mit denen zukünftige CI-Patient/-innen versorgt werden. Jedoch können auch langjährige CI-Nutzer/-innen von einer Umversorgung auf einen neuen Sprachprozessor profitieren, da diese fast immer mit den früher eingebrachten Implantaten kompatibel sind [4, 18]. Daher kann bei bereits mit einem CI versorgten Patient/-innen alle 6 Jahre die Umversorgung auf einen neuen Sprachprozessor geprüft werden. Da diese Umversorgung mit entsprechend hohen Kosten zulasten des Krankenversicherungsträgers verbunden ist, gilt es, den zusätzlichen Nutzen einer Umversorgung zu beweisen. Die Anforderungen des Medizinischen Diensts der Krankenkassen (MDK) zielen in diesem Versorgungsprozess vorzugsweise auf eine Dokumentation des audiologischen Nutzens ab [16]. Jedoch haben bereits frühere Untersuchungen gezeigt, dass der subjektive Nutzen der Patient/-innen im CI-Versorgungsprozess sich nicht vollumfänglich mit dem Teilaspekt der audiologischen Ergebnisse deckt [6, 26]. Um dieser Diskrepanz gerecht zu werden, wird im CI-Versorgungsprozess auch entsprechend der neuen Leitlinie zur CI-Versorgung zunehmend der Einsatz von Patient-Reported Outcome Measures (PROM), insbesondere von Lebensqualitätsmessinstrumenten, gefordert [8]. Für den Prozess der Sprachprozessorumversorgung hingegen existieren bislang noch keine standardisierten Empfehlungen zum Einsatz von PROM. Insbesondere bei Patient/-innen ohne Nachweis eines audiologischen Nutzens nach Erprobung eines neuen Sprachprozessors ist der Nachweis und die Dokumentation des subjektiven Mehrwerts notwendig, auch zur Argumentation gegenüber den Kostenträgern.

Das primäre Ziel der vorliegenden Studie war daher die standardisierte Dokumentation des subjektiven Nutzens der Sprachprozessorumversorgung bei erfahrenen CI-Nutzer/-innen nach Erprobung eines neuen Sprachprozessors in einem Zeitraum von 4 Wochen. Außerdem sollte der audiologische Nutzen der Umversorgung erfasst und mit dem durch die Patient/-innen berichteten Nutzen abgeglichen werden.

## Patient/-innen und Methoden

Die klinische Untersuchung wurde von der Ethikkommission der TU Dresden geprüft und genehmigt (BO-EK-251062020). Im Rahmen des routinemäßigen Umversorgungsprozesses wurden das audiologische und das subjektive Ergebnis mit neuem und altem Sprachprozessor bei bereits langjährig mit einem CI versorgten Erwachsenen im Erfassungszeitraum 2019–2022 analysiert.

Es wurde das Sprachverstehen in Ruhe und im Störgeräusch vor der Umversorgung mit dem alten Sprachprozessor (Testzeitpunkt 1) und nach 30-tägiger Testphase mit dem neuen Sprachprozessor (Testzeitpunkt 2) der Firmen MED-EL (Innsbruck, Österreich), Cochlear (Sydney, Australien) und Advanced Bionics (Valencia, USA) erfasst. An beiden Testzeitpunkten erfolgte zudem die Erfassung der subjektiven Höreinschränkung sowie der Zufriedenheit mit dem Sprachprozessor anhand von PROM.

### Audiologische Parameter

Die sprachaudiometrischen Untersuchungen erfolgten mit den klinischen Audiometern AT 900 und AT 1000 (Fa. Auritec, Hamburg, Deutschland) im Freifeld.

Zur Erfassung des Sprachverstehens in Ruhe wurde der Freiburger Einsilbertest im Freifeld mit neuem und altem Sprachprozessor bei 65 dB und 80 dB durchgeführt. Die Gegenseite war zum Testzeitpunkt unversorgt und wurde in Abhängigkeit vom Hörverlust vertäubt. Der Oldenburger Satztest (OlSa) wurde ebenfalls mit altem und neuem Sprachprozessor ohne Versorgung der Gegenseite durchgeführt. Die Messung erfolgte adaptiv bei 65 dB Störgeräusch und adaptivem Sprachschallpegel in der Konfiguration Signal und Störgeräusch von vorn (S0N0). Als Zielparameter wurde die Sprachverständlichkeitsschwelle ermittelt.

### Patient-Reported Outcome Measures

#### Abbreviated Profile of Hearing Aid Benefit

Der Abbreviated Profile of Hearing Aid Benefit (APHAB) wurde 1995 von Cox und Alexander als Weiterentwicklung des Fragebogens Profile of Hearing Aid Benefit (PHAB) veröffentlicht und ist das in der konventionellen Hörsystemversorgung am häufigsten eingesetzte Messinstrument zur subjektiven Einschätzung des Hörvermögens sowie zur Bewertung des Nutzens einer Hörsystemversorgung [7]. Das Messinstrument ist in 18 Sprachen verfügbar, wobei die deutschsprachige Version validiert und normiert wurde [15]. Die Patient/-innen sollen auf einer 7‑stufigen Skala angeben, in welchem Maße sie sich durch ihre Schwerhörigkeit in der geschilderten Situation beeinträchtigt fühlen. Geringere Werte gehen mit einer geringeren subjektiven Beeinträchtigung einher. Der Fragebogen umfasst 3 Skalen zur Bewertung des Hörvermögens in spezifischen Hörsituationen (EC-Skala, „ease of communication“, einfache Hörsituation ohne Nebengeräusche; BN-Skala, „background noise“, Hören mit Hintergrundgeräuschen; RV-Skala, „reverberation“, Hören in großen Räumen mit Echo oder Hallsituationen) sowie eine Skala zur Charakterisierung der Reaktionen auf Umweltgeräusche (AV-Skala, „aversiveness of sounds“, Hörempfinden von lauten Situationen).

#### Audio Processor Satisfaction Questionnaire

Der Audio Processor Satisfaction Questionnaire (APSQ) wurde zur Bewertung der Zufriedenheit mit dem aktuellen Sprachprozessor in deutscher Sprache entwickelt und validiert [5]. Der APSQ besteht aus 3 Subskalen (Comfort, Social Life, Usability) zu je 5 Items, welche anhand einer visuellen Analogskala von 0 bis 10 beantwortet werden. Die Items entstammen dem Hearing Implant Sound Quality Index (HISQUI) und der Speech, Spatial and Qualities of Hearing Scale (SSQ) [1, 10]. Höhere Werte gehen mit einer höheren Zufriedenheit der Patient/-innen einher.

#### Minimal Clinical Reported Difference

Für die Bewertung einer klinisch relevanten Verbesserung in den PROM wurde ankerbasiert die Minimal Clinical Reported Difference (MCID) ermittelt [2, 3, 13]. Der ankerbasierte Ansatz zur Bestimmung der MCID vergleicht die Veränderung innerhalb des PROM-Scores mit einem externen Kriterium, einem sog. Anker. Verwendet wurde hierzu ein Fragebogen, ein sog. Global Rating of Change (GRC), mit welchem die Patient/-innen die Veränderung ihres krankheitsspezifischen Zustands im Anschluss an die Sprachprozessorumversorgung bewerteten [13]. Als GRC wurde eine 10-Punkte-Skala genutzt mit einer Spannweite von −5 (stark verschlechtert) über 0 (unverändert) bis +5 (stark verbessert). Ergebnisse von > −1 bis < +1 wurden als keine bzw. unbedeutende Veränderung betrachtet und von der Auswertung ausgeschlossen. Ergebnisse von ≥ −2 bis ≤ −1 bzw. ≥ 1 bis ≤ 2 wurden als kleine Veränderung, gleichbedeutend mit der MCID, angesehen. Ergebnisse von ≥ −3 bis ≤ −2 bzw. ≥ 2 bis ≤ 3 wurden als moderate Veränderung, Ergebnisse von ≥ −4 bis ≤ −3 bzw. ≥ 3 bis ≤ 4 als große und Ergebnisse von ≥ −5 bis ≤ −4 bzw. ≥ 4 bis ≤ 5 als sehr große Veränderung angesehen [13]. Zur Berechnung der MCID wurden die PROM-Scores der Patient/-innen betrachtet, welche im GRC Ergebnisse von ≥ −2 bis ≤ −1 bzw. ≥ 1 bis ≤ 2 erzielt hatten. Der Durchschnitt der PROM-Score-Differenz aus der Messung vor und nach Sprachprozessorumversorgung ergab die MCID.

### Statistische Auswertung

Die statistische Auswertung erfolgte unter Einsatz von SPSS (Fa. IBM® SPSS®-Softwareplattform, Ehningen, Deutschland) und OriginPro 2022 (Fa. OriginLab, Northampton/MA, USA). Bei Normalverteilung wurde zum statistischen Mittelwertvergleich vor und nach Sprachprozessorumversorgung ein t‑Test angewendet. Das Signifikanzniveau wurde mit einem *p*-Wert ≤ 0,05 definiert. Als Kennwerte der deskriptiven Statistik sind Mittelwert und Standardabweichung angegeben.

## Ergebnisse

Es wurden Daten von 99 Patient/-innen (55,4 % weiblich und 44,6 % männlich) mit einem Durchschnittsalter von 60,6 ± 16,8 Jahren (Spannweite: 17–88 Jahre) analysiert.

Von diesen Patient/-innen waren 69 mit einem Implantat der Fa. MED-EL, 28 mit einem Implantat der Fa. Cochlear und 2 Patient/-innen mit einem Implantat der Fa. Advanced Bionics versorgt (Tab. 1).

**Table 1:** 

Hersteller	Sprachprozessor vor Umversorgung		Sprachprozessor nach Umversorgung	
Cochlear (*n* = 28)	Freedom	*n* = 1	CP950 (Kanso)	*n* = 2
	CP810	*n* = 13	CP1150 (Kanso 2)	*n* = 3
	CP910/920	*n* = 13	CP1000	*n* = 23
	CP950 (Kanso)	*n* = 1	–	–
MED-EL (*n* = 69)	DUET 2	*n* = 3	SONNET	*n* = 3
	OPUS 2	*n* = 42	SONNET 2	*n* = 41
	SONNET	*n* = 11	RONDO 2	*n* = 11
	RONDO	*n* = 13	RONDO 3	*n* = 14
Advanced Bionics (*n* = 2)	Q70	*n* = 2	Q90	*n* = 1
	–	–	M90	*n* = 1

### Sprachaudiometrische Ergebnisse

**Figure 1:**
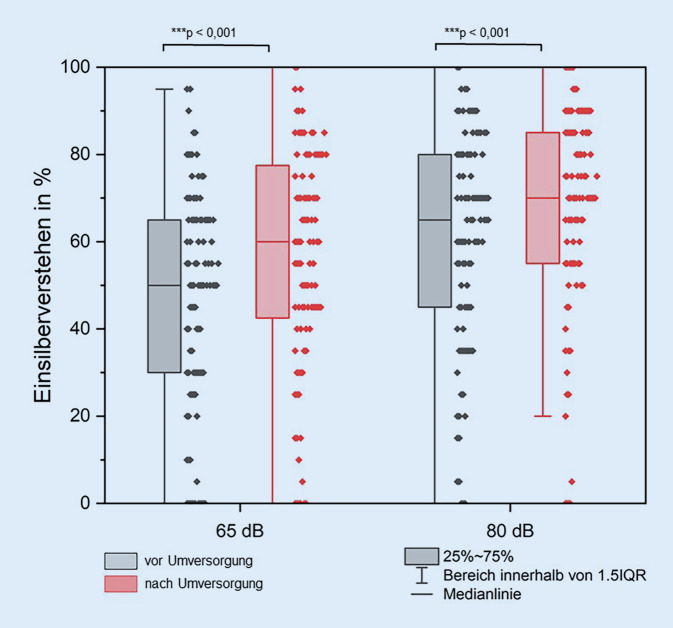

Mit dem neuem Sprachprozessor zeigte sich im Freiburger Einsilbertest in Ruhe bei 65 dB und 80 dB ein hochsignifikant besseres Sprachverstehen im Vergleich zur vorherigen Versorgung (mittlere Differenz 8,9 ± 25,9 Prozentpunkte bzw. 8,1 ± 29,7 Prozentpunkte, *p* < 0,001; Abb. 1). Im OlSa stellte sich eine signifikante Verbesserung der Sprachverständlichkeitsschwelle von 5,8 ± 13,2 dB S/N (Signal-Rausch-Abstand) auf 2,6 ± 7,3 dB S/N (mittlere Differenz 3,2 ± 10,7 dB S/N; *p* = 0,006) nach Sprachprozessorumversorgung dar.

### PROM

**Figure 2:**
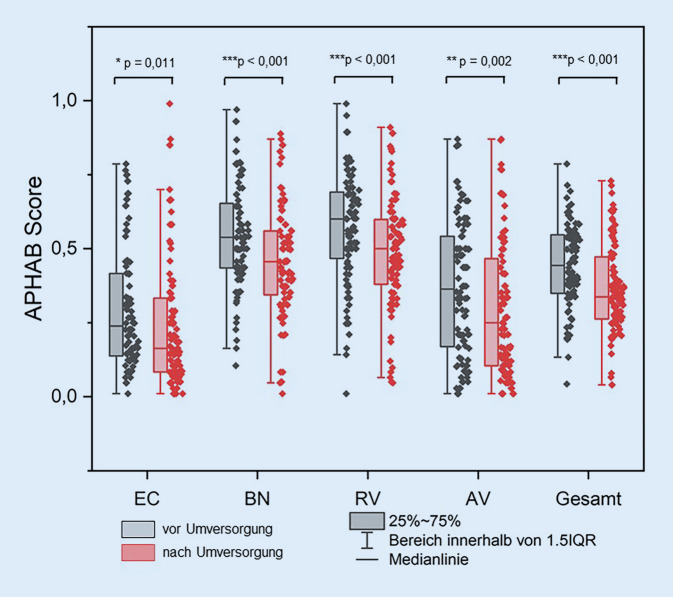

**Figure 3:**
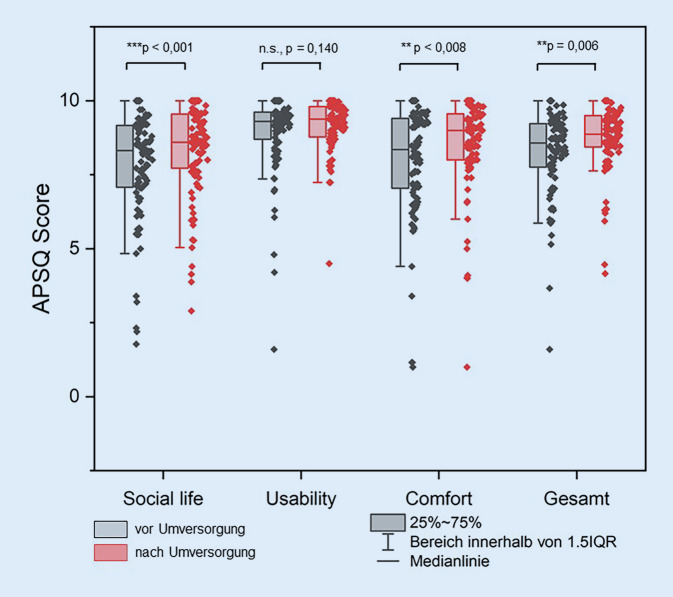

Bei der Bewertung des subjektiven Hörvermögens anhand des APHAB stellte sich für den Gesamtscore ein signifikanter (mittlere Differenz 0,07 ± 0,16; *p* < 0,001) subjektiver Nutzen durch die Sprachprozessorneuversorgung heraus (Abb. 2). In der Zufriedenheitsbewertung mittels APSQ zeigte sich für den Gesamtscore (mittlere Differenz: 0,42 ± 1,26; *p* = 0,006) sowie die Subskalen Social Life (mittlere Differenz: 0,54 ± 1,66; *p* = 0,001) und Comfort (mittlere Differenz: 0,57 ± 1,72; *p* = 0,008) ein signifikanter Versorgungsvorteil nach der Sprachprozessorumversorgung (Abb. 3).

### Abgleich der sprachaudiometrischen und der subjektiven Bewertung

Zur Bewertung einer sprachaudiometrischen Verbesserung durch den neuen Sprachprozessor wurde entsprechend der Hilfsmittel-Richtlinie ein Grenzwert von ≥ 20 Prozentpunkten im Freiburger Einsilbertest bei 65 dB bzw. eine Verbesserung von 2 dB S/N im OlSa festgelegt [11]. Ankerbasiert wurde für den APHAB-Gesamtscore eine MCID von ≥ 3,8 Prozentpunkten und für den Gesamtscore des APSQ von 0,74 Punkten ermittelt.

**Figure 4:**
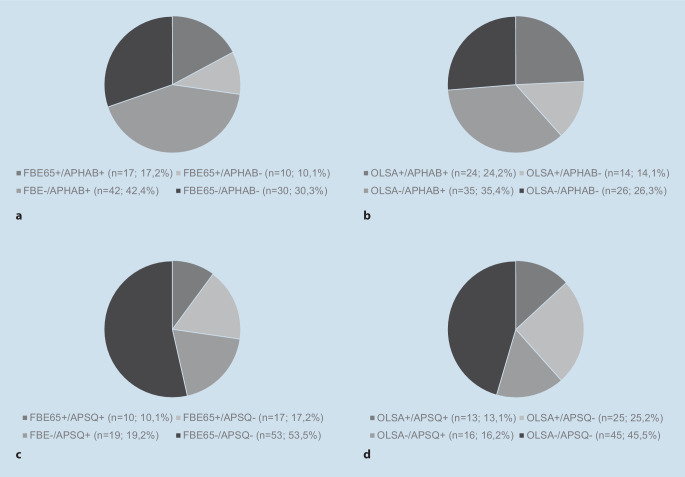

Bei gemeinsamer Betrachtung des Freiburger Einsilbertests und des APHAB-Gesamtscores zeigten sich für 47 Patient/-innen (47,5 %) einheitlich positive oder negative Ergebnisse, während 52 Patient/-innen (52,5 %) diesbezüglich inkongruente Ergebnisse aufwiesen. Bei 42,2 % der Patient/-innen zeigte sich im APHAB eine subjektive Verbesserung des Hörvermögens, obwohl sich im Freiburger Einsilbertest keine Verbesserung ergab (Abb. 4a).

Wurde die subjektive Zufriedenheit der Umversorgung anhand des APSQ mit den Ergebnissen des Freiburger Einsilbertests verglichen, ergaben sich bei 63 Patient/-innen (63,6 %) einheitlich positive oder negative Ergebnisse. Eine inkongruente Bewertung zeigte sich bei 36 Patient/-innen (36,4 %). Mithilfe des APSQ konnten 19 Patient/-innen (19,2 %) identifiziert werden, welche subjektiv von der Umversorgung profitierten, jedoch keine Verbesserung des Sprachverstehens im Störgeräusch aufwiesen (Abb. 4c).

Bei gemeinsamer Betrachtung der Ergebnisse im OlSa und im APHAB wiesen 50 Patient/-innen (50,5 %) einheitlich positive oder negative Ergebnisse auf, während sich bei 49 Patient/-innen (49,5 %) eine inkongruente Bewertung der Sprachprozessorumversorgung fand (Abb. 4b).

Auch bei der Analyse des Benefits anhand von OlSa und APSQ konnten insgesamt 41 Patient/-innen (41,4 %) mit differierenden Ergebnissen in der Sprachaudiometrie bzw. der subjektiven Beurteilung identifiziert werden (Abb. 4d).

## Diskussion

Die vorliegende Studie wurde so konzipiert, dass sie im Rahmen des routinemäßig durchgeführten Prozesses der Sprachprozessorumversorgung durchgeführt wurde. Zielstellung war es, den audiologischen Nutzen und die Wertigkeit der Integration von PROM in diesem Prozess der CI-Versorgung zu bewerten. Die Beurteilung einzelner Prozessoreigenschaften war hingegen nicht Zielstellung. Um einzelne Eigenschaften eines spezifischen Prozessors einer Implantatfirma zu bewerten, ist eine kontrollierte Untersuchung unter Berücksichtigung von Aspekten der Hörbiografie, Alter, Versorgungsituation der Gegenseite (z. B. bimodale Versorgung, einseitige Taubheit) und Implantationsjahr vonnöten. Die vorliegende Studie soll hingegen im Rahmen der kontinuierlichen Erfassung unter Einschluss aller Patient/-innen in einem festgesetzten Zeitraum anhand audiologischer und subjektiver Bewertungskriterien auf den tatsächlichen Nutzen der Sprachprozessorumversorgung für die einzelnen Patient/-innen im Alltag abzielen. Die in die Studie eingeschlossenen Patient/-innen weisen daher aufgrund der kontinuierlichen Erfassung und fehlenden Selektionskriterien eine hohe Heterogenität v. a. hinsichtlich Alter, Dauer des Hörverlusts, aber auch des Implantationszeitraums und der Implantatauswahl auf. In einer ähnlich heterogenen Gruppe mit 233 Erwachsenen und 261 Kindern/Jugendlichen wurde das Sprachverstehen vor und nach der Sprachprozessorneuversorgung gegenübergestellt [23]. In der Erwachsenengruppe zeigte sich eine signifikante Verbesserung im Sprachverstehen um 17,5 %, in der Gruppe von Kindern und Jugendlichen um 10 %.

Bisherige Studien haben stets eine signifikante Verbesserung des audiologischen Nutzens für jede Sprachprozessorgeneration ergeben. Da beim Vergleich unterschiedlicher Prozessorgenerationen der Abgleich der Patient/-innen hinsichtlich der Hörparameter und demografischen Daten schwierig ist, wurde der Vergleich unterschiedlicher Prozessoren oft im Rahmen des klinischen Umversorgungsprozesses erhoben, sodass die evaluierte Gruppe der Patient/-innen auch als eigene Kontrollgruppe in die jeweilige Untersuchung einging. Die Mehrzahl der bisherigen Studien bewertet dabei den Versorgungsvorteil eines speziellen Prozessors einer Implantatfirma. So wurde in klinischen Studien der Vorteil der Umversorgung auf den CP810 [18], den C910 [4, 20, 22, 25], den CP1000 [27], den Rondo [17] und den Opus 2 [14, 24] bestätigt. Einzelne Untersuchungen bewerten hingegen explizit einzelne technologische Neuerungen eines neuen Sprachprozessors im Rahmen der Neuversorgung, beispielsweise den Effekt spezifischer Richtmikrofonmodi [19], Signalvorverarbeitungsstrategien [21] oder der Verbindungsfähigkeit des Sprachprozessors mit externen Geräten [27].

Aufgrund der in den einzelnen Studien divergierenden Messmethodik hinsichtlich des verwendeten Sprachmaterials und der genutzten Prüfsprachschallpegel ist eine studienübergreifende Bewertung des Nutzens der Sprachprozessorumversorgung kaum möglich. Anhand der derzeitigen Studienlage findet sich für einsilbige Prüfwörter bei 60–65 dB SPL im Mittel eine Verbesserung um 3–15 % [4, 14, 18, 23, 24]. Bei der Messung mit mehrsilbigen Zahlen wurde in bisherigen Untersuchungen keine signifikante Verbesserung durch die Umversorgung ermittelt, was an dieser Stelle auf einen Deckeneffekt zurückzuführen ist [14]. Bei der Bewertung der Verbesserung des Sprachverstehens ist jedoch nicht nur die gemittelte Zunahme an Sprachverstehen von Bedeutung, sondern auch der Anteil an Patient/-innen, bei denen eine klinisch relevante Verbesserung des Sprachverstehens anhand audiologischer Messwerte offensichtlich wird. An dieser Stelle wäre eine statistisch begründete MCID für den Freiburger Einsilbertest hilfreich. In der klinischen Routine orientiert man sich meist an der aus der konventionellen Hörsystemversorgung übernommenen und in der Hilfsmittel-Richtlinie hinterlegten Differenz von 20 % [11]. Wie viele Patient/-innen tatsächlich eine Verbesserung des Sprachverstehens um 20 % verzeichnen, wird in bisherigen Studien nur sporadisch dokumentiert. So findet sich in der Untersuchung von Mosnier et al. bei 37 % der analysierten 35 Patient/-innen eine Verbesserung um mindestens 20 % [18]. Insbesondere bei Patient/-innen mit gutem Sprachverstehen ist bei den Messungen in Ruhe mit einem Deckeneffekt zu rechnen, sodass bei diesen Patient/-innen audiologisch kein zusätzlicher Nutzen durch die Sprachprozessorumversorgung messbar wird. Beispielsweise zeigten Seebens und Diller bei der Bewertung der Umversorgung auf den Opus 2 bei Patient/-innen mit bereits sehr gutem Sprachverstehen eine geringere Verbesserung (7 %) im Sprachverstehen gegenüber der Gruppe aller analysierten Patient/-innen (10 %) [24]. Auch bei Patient/-innen, die nach Abschluss der CI-(Re‑)Habilitation kein Sprachverstehen entwickelt haben, ist die Ermittlung eines audiologischen Mehrwerts durch die Umversorgung nur schwer möglich, sodass sich gerade bei diesen Patient/-innen der Einsatz psychometrischer Messinstrumente zur Erfassung des subjektiven Nutzens anbietet.

Die audiologischen Kriterien, die von den Krankenkassen bei der Bewertung der Notwendigkeit einer Umversorgung zugrunde gelegt werden, orientieren sich an den Abnahmekriterien der konventionellen Hörsystemversorgung. Beispielsweise wird als Erfolgskriterium eine Verbesserung des Sprachverstehens um −2 dB S/N im OlSa gefordert. Durch die Anwendung von Satztests im Störgeräusch wird die Abbildung der audioverbalen Alltagskommunikation angestrebt. Der OlSa kann jedoch nur bei Patient/-innen durchgeführt werden, die mit dem CI ein Sprachverstehen erreichen. Eine studienübergreifende Bewertung der Sprachprozessorumversorgung ist derzeit aufgrund der divergierenden Messmethodik und Berichtserstattung selbst im deutschsprachigen Raum nicht möglich. Tendenziell zeigte sich, analog zu der vorliegenden Untersuchung, eine Verbesserung des Sprachverstehens im Störgeräusch [4, 12, 18–20, 23, 24], wobei berücksichtigt werden muss, dass in einzelnen Studien explizit auf die Bewertung spezieller Störgeräuschunterdrückungsalgorithmen oder Richtmikrofonmodi lag [12, 19]. Um den Vorteil dieser neuen Entwicklungen in den einzelnen Sprachprozessorgenerationen zu erfassen, kann es hilfreich sein, die Messmethodik bzw. -anordnung zur Erfassung des Sprachverstehens im Störgeräusch anzupassen. So wird beispielsweise der Mehrwert von Richtmikrofonen erst bei der Messung mit seitlichem Störgeräusch deutlich [12].

Im Rahmen einer Hörsystemüberprüfung nach der Hilfsmittel-Richtlinie kommt in Deutschland seit Jahren der APHAB-Fragebogen obligatorisch zum Einsatz. Mithilfe des APHAB lassen sich gezielt Defizite mit therapeutischer Dimension sowohl für einzelne Patient/-innen, aber auch für die Weiterentwicklung des Hörsystems herausarbeiten. Die Validierung des deutschsprachigen APHAB liegt nur für dessen Verwendung im Versorgungsprozess mit konventionellen Hörsystemen vor. Aufgrund seiner internationalen Verfügbarkeit wird der APHAB jedoch auch zunehmend zur Bewertung der CI-Versorgung genutzt. Der APHAB fokussiert auf die Erfassung der physischen Domäne der Höreinschränkung und bietet sich daher an, das Hörvermögen mit altem und neuem Sprachprozessor aus der Sicht der Patient/-innen zu bewerten. Für alle 4 Hörsituationen zeigte sich aus der Perspektive der Patient/-innen eine signifikante Verbesserung des Hörvermögens durch die Sprachprozessorumversorgung. Klinische Untersuchungen zum subjektiven Nutzen der Umversorgung liegen derzeit nur sporadisch vor. Mosnier et al. zeigten eine Verbesserung beim Hören mit Hintergrundgeräuschen und halliger Umgebung bei Umrüstung auf den CP810, jedoch keinen subjektiven Nutzen bei Umrüstung auf den CP 910 anhand des APHAB [18].

Um weitere für die Patient/-innen relevante Aspekte des Sprachprozessorumversorgung abzudecken, ist daher ein weiteres Messinstrument heranzuziehen. Insbesondere, um Tragekomfort und Handhabung eines Sprachprozessors zu bewerten, wurden mitunter durch Arbeitsgruppen eigene Fragebögen, teilweise spezifiziert für einen spezifischen Sprachprozessor, konzipiert, welche aufgrund des fehlenden Nachweises von Validität und Reliabilität nicht für einen verbreiteten Einsatz empfohlen werden können. Anhand dieser Fragelisten zeichnete sich ein Versorgungsvorteil hinsichtlich des Tragekomforts, der Bedienung und der Hörbeeinträchtigung mit den neuen Prozessoren ab [14]. Da diese Fragelisten meist für einen spezifischen Sprachprozessor entworfen wurden, ist deren Anwendung beim Vergleich unterschiedlicher Sprachprozessorgenerationen nicht zweifelsfrei möglich. Diese methodische Lücke konnte durch die Bereitstellung des in deutscher Sprache entwickelten und validierten APSQ geschlossen werden [5]. Obwohl sich in der bereits publizierten Untersuchung zur Validierung des APSQ ein Deckeneffekt abzeichnete [5], konnten in der vorliegenden Studie signifikante Unterschiede im Gesamtscore sowie den Subdomänen zwischen alten und neuen Sprachprozessor identifiziert werden. Die größte methodische Unschärfe ergibt sich beim Einsatz psychometrischer Messinstrumente, aber auch in Sprachaudiometrie als psychoakustisches Testverfahren durch mögliche Verzerrungen, da Patient/-innen die Testverfahren mit dem Wissen durchführen, den neuen Sprachprozessor nur bei Nachweis von dessen Überlegenheit zu erhalten. Aufgrund der Studienmethodik und des klinischen Routineprozesses zur Bewertung des Nutzens der Sprachprozessorumversorgung ist jedoch eine randomisierte verblindete Testung nicht möglich.

Neben der standardisierten Erfassung der subjektiven Hörbeeinträchtigung und der subjektiven Zufriedenheit mit den jeweiligen Sprachprozessoren bieten sich beide Messinstrumente jedoch auch dazu an, Patient/-innen zu identifizieren, bei denen sich audiometrisch kein zusätzlicher Nutzen durch die Sprachprozessorumversorgung abzeichnet, aber subjektiv ein relevanter Nutzen geschildert wurde. In der vorliegenden Untersuchung konnte ein Anteil an Patient/-innen von 35–42 % identifiziert werden, der in den audiologischen Untersuchungen keinen Zugewinn an Sprachverstehen verzeichneten, jedoch subjektiv eine relevante Verbesserung des Hörvermögens angab. Um gegenüber den Kostenträgern an dieser Stelle die Umversorgung begründen zu können, ist der Einbezug psychometrischer Messinstrumente in diesem Teilaspekt der CI-Versorgung weiter zu unterstützen, da audiologische Messungen allein den Nutzen der Patient/-innen nur unzureichend widerspiegeln.

Da die Bewertung spezieller Prozessoreigenschaften nicht Gegenstand der vorliegenden Analyse war, bleibt aktuell noch unklar, welche spezifischen Merkmale neuer Sprachprozessorgenerationen zum subjektiven Vorteil einzelner Patient/-innen beitragen. Da keine Kontrollgruppe gewählt wurde, lässt sich ein hochrelevanter Placeboeffekt nicht ausschließen. In Folgeuntersuchungen mit entsprechender Kontrollgruppe gilt es, dessen Ausmaß zu identifizieren, um die Ergebnisse der PROM auch als wissenschaftlich fundierte Argumentationsgrundlage verwenden zu können.

Die derzeit in den Begutachtungsrichtlinien des MDK [16] hinterlegte Forderung nach einer „erheblichen“ Verbesserung des Sprachverstehens in Ruhe bzw. im Störgeräusch durch die Sprachprozessorumversorgung zur Gewährung der Neuversorgung spiegelt aktuell nur einen sehr begrenzten Blick der Kostenträger auf diesen Versorgungsprozess wider. Eine schlüssige sozialmedizinische Beurteilung als Grundlage für die Entscheidung der Leistungserbringer erfordert einen Abgleich von beantragtem und gutachtlich festgestelltem Versorgungsbedarf, welcher sich bei der Sprachprozessorumversorgung nicht allein anhand audiologischer Kenndaten festmachen lässt. Gerade die Kombination unterschiedlicher Bewertungsebenen unter Berücksichtigung validierter audiologischer und psychometrischer Messinstrumente sollte entsprechenden Umversorgungsanträgen zukünftig ein stärkeres Gewicht im Sinne der Patient/-innen verleihen.

## Fazit für die Praxis

- Die Versorgung mit einem neuen Sprachprozessor führt bei Cochleaimplantat(CI)-Patient/-innen zu einer Verbesserung des Sprachverstehens in Ruhe und im Störgeräusch sowie zu einer Optimierung der subjektiven Hörbeeinträchtigung und der Zufriedenheit der Patient/-innen.
- Mithilfe von Patient-Reported Outcome Measures (PROM) können Patient/-innen identifiziert werden, welche subjektiv von der Sprachprozessorumversorgung profitieren, jedoch keine Verbesserung in den sprachaudiometrischen Untersuchungen aufweisen.
- PROM sollten in den Routineprozess der Sprachprozessorumversorgung integriert werden, um eine umfassende, individuelle Bewertung des Versorgungsnutzens herauszuarbeiten.
- PROM sollten zusätzlich zu sprachaudiometrischen Daten eine Argumentationsgrundlage bei der Beantragung der Kostenübernahme für die Umversorgung bei den Kostenträgern darstellen.
